# Pedagogical innovation to promote physical activity in pregnancy: Interprofessional and real-life settings on the example of the educational program Move Your Baby

**DOI:** 10.18332/ejm/146629

**Published:** 2022-05-25

**Authors:** Mathilde Hyvärinen, Franziska Schläppy, Claire de Labrusse, Jennifer Wegrzyk

**Affiliations:** 1 Department of Research, HESAV School of Health Sciences, HES-SO University of Applied Sciences and Arts Western Switzerland, Lausanne, Switzerland; 2 Department of Health Promotion and Prevention, University Center for Primary Care and Public Health (Unisanté), Lausanne, Switzerland; 3 Department of Midwifery, HESAV School of Health Sciences, HES-SO University of Applied Sciences and Arts Western Switzerland, Lausanne, Switzerland

**Keywords:** pregnancy, education, students, physical activity, midwifery, health promotion

## Abstract

**INTRODUCTION:**

Despite scientific evidence on health benefits of an active lifestyle during and after pregnancy, a gap still exists between current and recommended practice in physical activity counselling. Undergraduate education in midwifery is fundamental for physical activity promotion in professional practice. The aim of this article is to present pedagogical aspects, preliminary results and discuss the relevance of the educational program Move Your Baby.

**METHODS:**

Between 2018 and 2020, 23 midwifery students (BSc) participated in the program at the School of Health Sciences (HESAV), HES-SO University of Applied Sciences and Arts Western, Lausanne, Switzerland. Theoretical and practical workshops as well as adapted physical activity sessions, in direct contact with pregnant women, were offered and supervised by professional midwives and one expert in adapted physical activity. Data analysis based on an exploratory self-administered questionnaire was performed to rate pedagogical effectiveness, perceived skill level and identify barriers and facilitators to promote physical activity in their future profession.

**RESULTS:**

Midwifery students perceived improvement in their knowledge, skills and confidence to promote physical activity during pregnancy. They rated the program as pedagogically effective. However, several barriers were identified such as lack of time and material resources to promote physical activity in professional practice.

**CONCLUSIONS:**

This community-oriented educational program based on interplay of theory, hands-on experience and interprofessional collaboration was rated successful. Teaching physical activity in real-life settings facilitates midwifery students to identify with their professional role in the field of health promotion. Midwifery students require more opportunities to promote physical activity in their professional practice.

## INTRODUCTION

Over the past three decades, rates of complications such as gestational diabetes, preeclampsia, gestational hypertension, and macrosomia, have strongly increased, mainly due to an increased sedentary lifestyle in pregnancy^1^ and maternal obesity^2^. Physical activity (PA) during pregnancy is strongly recommended. An active lifestyle improves health of both mother and child, and notably reduces obstetrical complications and the risk of developing non-communicable diseases in adulthood^3,4^. International guidelines recommend 150 minutes of moderate-intensity PA per week during pregnancy^5^. Pregnancy is an opportune time for the promotion of healthy lifestyles because health behavioral determinants evolve naturally; women are getting in regular contact with healthcare professionals (HCPs), and are more likely to adopt a healthy lifestyle if recommended^6^. PA promotion should be addressed in an interprofessional approach between gynecologist, midwives, nurses, physiotherapist and exercise professional^3,7^. Midwives are ideally placed as they deliver woman-centered care during pregnancy^8^. They can act as mediator between pregnant women and other HCPs to promote PA and should be familiar with the content of PA recommendations and basic exercise training. However, midwives lack either knowledge of existing recommendations and pregnancy-related benefits or resources, and confidence to adequately address the topic^7,8^. Since the publication of the Swiss guidelines on PA during pregnancy and postpartum in 2018^9^, the School of Health Sciences, Lausanne (HESAV) intensified PA promotion in the midwifery Bachelor’s program. In collaboration with the Center for Primary Care and Public Health, Lausanne (Unisanté), the educational program *Move Your Baby* (MYB) was designed to create an interactive environment for midwifery students and pregnant women. The goal of this program was to empower and prepare midwifery students to actively promote PA in pregnancy. This article aims to present the pedagogical aspect, preliminary results and discuss the relevance of an innovative, community-oriented educational program to promote PA in pregnancy.

## METHODS

### Participants and program overview

The MYB program is based on active learning strategies^10^ and interprofessional guidelines^3^. The PA level of HCPs impacts on confidence in counselling women to promote PA^11^. Therefore, the MYB program was designed to empower students in PA by participating and delivering adapted PA sessions^12^. Adapted PA sessions were ‘multicomponent PA’, i.e. combining different types of exercise (mainly pelvic floor exercises, aerobic endurance, muscle strengthening, and balance training) in a structured setting^5^. Since the program MYB was launched at HESAV in 2018, more than 100 midwifery Bachelor’s students attended a basic (mandatory) training on PA promotion in pregnancy comprising 8 hours over 2 years (5.5 h in first year and 2.5 h in second year). Theoretical class (3 h) was taught by a midwifery teacher, and included knowledge on physiological adaptations, PA recommendations, health benefits and midwives’ role. Two workshops (1.5 h each) were taught by an exercise professional (MSc in Sports Science, Unisanté) and included skill acquisition on key messages in PA promotion and adapted PA. In the first year, students per group of 3 participated with pregnant women in an adapted PA session held by an exercise professional. In the second year, students carried out an adapted PA session per group of 3 in direct contact with pregnant women under the supervision of an exercise professional and a midwifery teacher. Eleven students participated in an advanced (optional) module in PA promotion held in the second year including 10 additional hours of workshops and 4 additional adapted PA sessions to deepen all the topics studied in the basic module. An adapted PA session (1 h) was structured as follows: a fun warm-up; a main part including strengthening, balance and endurance exercises, and a cool-down with mobility and stretching exercises. Each exercise was presented with 3 alternatives of performing while adapting intensity according to the individual physical fitness level and daily condition of women. Based on international guidelines^5^, messages on PA promotion during daily life were delivered throughout each session. All adapted PA sessions were free-of-charge and took place once per week through the entire school year in the school facilities during the lunch break.

### The program’s educational objectives

At the end of the program, the participating midwifery students should feel confident to actively promote PA in pregnancy. The fixed objectives were: 1) to acquire and provide evidence-based counselling based on valid national and international recommendations for PA in pregnancy^3^, 2) to conceive adapted PA session while adapting to individual needs, 3) to teach the adapted PA sessions, 4) to experience PA and interprofessional context, and 5) to argue the role of midwifery profession in PA promotion.

### Evaluation of the program

An exploratory self-administered survey based on Gérard’s method^13^ and adapted to the context was conducted before and after the program to obtain preliminary results on pedagogical effectiveness of MYB program. Students (n=23) of Bachelor’s in years 2018–2020 were included in this exploratory approach that aimed at evaluating students’ perception on skill acquisition. Information about perceived students’ skill level was evaluated for each of the above-listed objectives using a Likert scale (0–5)^13^. Pedagogical effectiveness was assessed according to three dimensions: perceived relevance of fixed objectives, skill acquisition in PA promotion, and transferability in practice likewise rated on a Likert scale (1–4)^13^. Moreover, the formulation of open questions as part of the questionnaire allowed for identification of barriers and facilitators to promote PA as perceived by students and analyzed using the COM-B model^12^. The survey was conducted within the scope of reflective practice and students participated on a voluntary basis.

## RESULTS

In all, 23 midwifery students received and completed mandatory basic training out of which 5 attended in addition the optional advanced module. Of the 23 students, 17 completed the self-administered surveys. Students reported increased knowledge, skills and confidence in promoting PA according to the pedagogical objectives (mean score: 4/5). The interaction with pregnant women enabled them to transfer their theoretical knowledge into practice. According to the students, the pedagogical effectiveness of the MYB program was high (mean score: 3.35/4). However, midwifery students stated several barriers to promote PA in pregnant women as experienced during internships and their curriculum. As a main barrier, they experienced a lack of resources, notably time and social support from midwives during their internships to correctly apply acquired knowledge and skills in current professional settings. Students that participated in the advanced module perceived less barriers and felt more competent and legitimate to promote PA than those students that participated in the basic module (Table 1).

**Table 1:** Barriers to promote physical activity in pregnant women identified by midwifery students in the pedagogical program Move Your Baby of HESAV, School of Health Sciences, Lausanne (2018–2020) according to the COM-B Model12 (N=17)

*Factors*	*Barriers*
**Capability**	
Physical	
Psychological	Lack of confidence, omission to talk about PA during consultations
**Opportunity**	
Physical	Lack of time and material resources, not enough practice in the training program
Social	Midwives do not promote PA. Lack of social support from midwives
**Motivation**	
Non-automatic	PA promotion perceived as irrelevant for the profession
Automatic	Feeling uncomfortable addressing the topic with women, fear of not practicing and losing skills

PA: physical activity.

## DISCUSSION

As a community-oriented educational program, MYB offers a unique opportunity for interprofessional collaboration and for midwifery students to improve their competence in PA promotion. At the same time, pregnant women could benefit from free-of-charge sessions and professional guidance. Preliminary results indicate pedagogical positive impact of the program with enhanced knowledge, skills and confidence to actively promote PA in future professional practice. Students reported several barriers to promote PA such as lack of time, material resources and social support from midwives.

### Challenges for midwives promoting physical activity

As stated by Walker et al.^7^, education promotes a change in attitudes and values influencing identity, role, motivation, and beliefs. Thus, combined evidence-based theory, active hands-on learning and adapted PA session seem to support students to feel more responsible and legitimate to promote PA during pregnancy, as students that participated in the advanced module stated less barriers than those who completed only the basic module. Student participants reported difficulties to apply their newly acquired skills during their internships mainly due to a lack of opportunity in current professional environments. A lack of environmental opportunities are recurrent barriers cited by professionals in the literature such as lack of time, leaflets, infographics, available classes, and referral pathways^8,14,15^. A lack of credible models due to the gap between current and recommended practice in PA counselling^6,7,8^ hinders students from learning by imitation. Walker and al.^7^ assume that improved educational aspects in curriculum, on-the-job training, and advanced training would support the development of skills, knowledge and self-efficacy of midwifery students and professionals to actively promote PA among pregnant women. Midwives are not expected to be unique providers of PA guidelines in pregnancy. Therefore, midwives should know when, how, and towards whom to guide women in need of specific support^7,15^. In addition to the above-mentioned educational aspects, De Vivo and Mills^14^ also mention to support midwives in PA promotion, and a need to improve access, availability and awareness of suitable activities in the local community such as free adapted PA sessions. Perceived barriers in PA promotion should be taken into consideration not only by pedagogical institutions but also by healthcare institutions and health policies to enhance positive interprofessional work environments for PA promotion with continuing education, material resources and suitable activities in the local community.

### Limitations

Several limitations should be acknowledged. The sample of students was small and has been affected by the COVID-19 pandemic. As the program is ongoing, the preliminary results presented here will be confirmed by a larger sample in the future. A randomized controlled and longitudinal study design is needed to assess the effectiveness of the program in terms of students’ behavioral change and causality.

## CONCLUSIONS

MYB program, a pedagogical innovation for midwifery students, seems to be promising to empower and prepare midwifery students to actively promote PA to women during their pregnancy. Students highlighted the importance of interprofessional teaching and direct contact with pregnant women to acquire, apply and transfer knowledge, skills and confidence into professional practice. However, they identified several barriers such as lack of time and material resources to promote PA during pregnancy in professional practice.

## Data Availability

The data supporting this research are available from the authors on reasonable request.
